# Hepatitis C virus reinfection among people who inject drugs in the country of Georgia and injection-related risk factors: Implications for HCV elimination in the EECA region

**DOI:** 10.1371/journal.pone.0330764

**Published:** 2025-09-08

**Authors:** Liana Shengelaia, Emeli J. Anderson, Maia Butsashvili, Lasha Gulbiani, Giorgi Kanchelashvili, Jack Dehovitz, Mamuka Djibuti, Tomoko Udo, George Kamkamidze

**Affiliations:** 1 Ivane Javakhishvili Tbilisi State University, Faculty of Medicine, Tbilisi, Georgia; 2 Health Research Union and Clinic NeoLab, Tbilisi, Georgia; 3 SUNY Downstate Health Sciences University, Department of Medicine, Brooklyn, New York, United States of America; 4 Partnership for Research and Action for Health, Tbilisi, Georgia; 5 The College of Integrated Health Sciences, University at Albany, New York, United States of America; Institute of Clinical and Experimental medicine: Institut klinicke a experimentalni mediciny, CZECHIA

## Abstract

Hepatitis C (HCV) infection is a major global health challenge, with particularly high prevalence among people who inject drugs (PWID) in the Eastern European and Central Asian region (EECA). While the country of Georgia has made major progress in reducing overall HCV prevalence, less is known about HCV reinfection rates and risk factors for reinfection among PWID. In this study, we aimed to: (1) estimate HCV reinfection rates and (2) identify risk factors associated with HCV reinfection among PWID. Data were from the 2022 Integrated Bio-Behavioral Surveillance Study (IBBS) of PWID in Georgia. For the present analysis, the sample was restricted to the 462 PWID who had previously received HCV treatment through the national elimination program. The survey included a behavioral component (face-to-face interviews using a standardized questionnaire covering injection-related risk behaviors), and a laboratory component (blood samples for HCV RNA testing). We conducted regression models based on bivariate analyses to identify risk factors associated with laboratory-confirmed HCV reinfection. Overall, the reinfection rate was 13% among PWID in our sample. We found that younger PWID had higher odds of HCV reinfection compared to older PWID. The highest reinfection rate was among participants aged 18–24, (33.3%), although this group comprised a small portion of the sample (n = 3). Those reporting public injecting, had an adjusted odds ratio AOR of 8.08 [95% CI: 2.13,30.98] of HCV reinfection. At the time of the study, continuous opioid injection every day during the last 12 months was also associated with reinfection with an AOR of 2.26 [95% CI: 1.06,5.01]. Reinfection presents a challenge to HCV elimination. We identified several key factors that may influence reinfection including age, injection environment, and duration of drug injection. These findings emphasize the necessity for an integrative approach to harm reduction that addresses both behavioral and environmental risk factors.

## Introduction

Hepatitis C virus (HCV) is a major public health problem worldwide. In 2022, the World Health Organization (WHO) estimated that there were 1 million new cases of hepatitis C virus (HCV) globally and approximately 58 million people living with chronic HCV infection [1]. People who inject drugs (PWID) bear a disproportionate burden of HCV, representing a quarter of new HCV infections and almost 40% of chronic infections [2]. PWID in Eastern European and Central Asian countries (EECA) are particularly impacted, with an estimated 1.5 million PWID living with HCV in the Eastern European region [3]. While high rates of primary infection are well documented, less is known about HCV reinfection rates and associated risk factors in the region. The prevention of reinfection is critical to achieve HCV elimination by 2030 both within the EECA region and globally as suggested by the WHO [4].

The Eastern European country of Georgia has a high burden of injection drug use (IDU) and HCV infection [5]. Additionally, Georgia was the first country to implement a national Hepatitis C elimination program, instituted in 2015 with the goal to decrease the HCV prevalence by 90% nationwide by 2020 through providing universal access to harm reduction services, extensive screenings, linkage to care services, and treatment services [5]. Between 2015 and 2021, the prevalence of chronic HCV decreased by 67% (from 5.4% in 2015 to 1.8% in 2021) at the national level [6]. HCV antibody positivity was detected in 58.1% of the study population among PWID at the beginning of intervention. Geographic variation was observed, with the city of Zugdidi showing the highest anti-HCV prevalence at 76.0%, while the city of Kutaisi demonstrated the lowest at 46.0%. In addition, upon verification with the national HCV treatment database, 19.4% of participants who self-reported receiving HCV treatment had no documented treatment history [7]. HCV treatment uptake is high among PWID in Georgia, with 93.4% of individuals also receiving Opioid Substitution Therapy (OST) at integrated treatment centers [8]. Another study with PWID who completed HCV treatment through the national elimination program, however, found that reinfected individuals reported recent injection at higher rates than those who were not reinfected (79.5% vs. 65.4%), suggesting increased risk for reinfection among those who resumed IDU [9].

Given the high prevalence coupled with widespread IDU, HCV reinfection among PWID may pose a significant threat to HCV elimination. Research is needed to understand the impact and risk factors associated with HCV reinfection.

Previous research has identified several risk factors for HCV reinfection among PWID, including ongoing drug use, drug treatment, sharing needles, and having multiple partners [10–14]. Ongoing drug use has been shown to significantly increase the risk of HCV reinfection after successful treatment, although with varying rates ranging from 1.9 cases per 100 person-years to 15.5 cases per 100 years [10–16]. Opioid Substitution Treatment (OST) may help attenuate reinfection rates; for example, a recent meta-analysis estimated a reinfection rate of 0.55 cases per 100 person-years among those receiving OST [15]. Sharing needles was also identified as predictive of reinfection in this study. In addition to IDU, recent injecting drug use (in the previous 6 months) and having multiple injecting partners have also been shown to predict the risk of reinfection [17]. A higher rate of HCV reinfection was observed among PWID with shorter follow-up, suggesting higher reinfection risk early post-HCV treatment [18]. In one cohort study, the results showed a significant decrease in HCV reinfection with increasing post-sustained virologic response (SVR) follow-up [19]. These findings underscore the importance of post-SVR interventions, including regular follow-up visits, counseling services, and early identification of reinfection risk factors.

The present study evaluated the association between injection-related risk behaviors and HCV reinfection among PWID in Georgia, using data from the 2022 Integrated Bio-Behavioral Surveillance study (IBBS) [20]. Our goal was to understand risk behaviors among PWID and their contribution to HCV reinfection. Results will help inform strategies to develop prevention, counseling, and treatment services specifically to address risk of reinfection among PWID populations.

Hypothesis: Using a needle or syringe previously used by someone else is at higher risk of HCV reinfection compared to those who reuse their own needles.

## Methods

The Integrated Bio-Behavioral Survey (IBBS) among PWID in Georgia was conducted between May and June 2022. Data were collected from seven major cities resulting in the following sample sizes: 381 in Tbilisi, 270 in Gori, 269 in Telavi, 275 in Zugdidi, 271 in Batumi, 269 in Kutaisi, and 270 in Rustavi. Respondent-driven sampling (RDS) was used to sample participants. Briefly, an initial 3–7 participants were selected in each city (the “seeds”) based on age, sex, connection to different groups of PWID, and residential area. The seeds then recruited an additional three participants using provided coupons, who in turn recruited another three, and so on until the desired sample size was reached (n = 2005 across all seven cities). Participants were eligible if they were age 18 or older, had practiced drug injection in the 30 days before the survey, resided in one of the selected cities, were able to complete the interview in Georgian, consented to participate in both components of the study (questionnaire completion and testing), and gave written informed consent [20]. This study is a secondary analysis of de-identified data collected through the IBBS surveys, were were approved by the relevant institutional ethics review boards.

For this analysis, we restricted our sample to those individuals with a history of HCV infection who achieved SVR after treatment (n = 462). To confirm both, we used data from the national hepatitis C virus (HCV) elimination program and cross-referenced it with the IBBS data. Statistical analyses were performed in R version 4.4.1 and SPSS version 21.

### Behavioral survey

Data was collected via face-to-face interviews with participants, including data on demographics, alcohol use, drug use history, drug use-related behaviors, sexual history, knowledge and attitude towards HCV and HIV/AIDS, prevention programs, and social networks. Our primary outcome was HCV reinfection, and key demographic and drug use-related variables were included as covariates. Demographic and general behavioral variables included city of residence (one of the seven included in the study), age, gender, education, employment status, marital status, cohabitation status (with whom the participant lives), previous incarceration, and alcohol use. Drug-related variables included who the participant injects with, the type of drug injected in the month before the survey, injecting frequency, needle reuse, use of prefilled needles, injection environment (e.g., in an automobile), and receipt of drug treatment in the past year.

### Biomarker component

Blood tests were done for HIV, hepatitis B virus, and HCV infections. To determine if someone was infected with HCV, we used rapid or Enzyme-linked Immunosorbent Assays (ELISA) testing followed by confirmatory testing. We screened for anti-HCV (HCV antibodies) using On Site HCV Ab Plus Combo Rapid tests (CTK Biotech) or ELISA for HCV Ab – CVAB using Diagnostic BioProbes Srl- Dia-pro. Polymerase Chain Reaction was used to detect HCV RNA for confirmation of anti-HCV positive cases using HCV Real-TM Quant Dx (Sacace Biotechnologies).

### Statistical analysis

Descriptive analyses were conducted on all covariates, including the overall distribution of each variable and stratified by HCV reinfection status. We used chi-square tests for categorical and t-test or Fisher’s exact test for continuous variables to evaluate the bivariate association between the covariates and HCV reinfection. Logistic regression was used to calculate the odds ratio (OR) and 95% confidence intervals (CIs) to first evaluate the association of each covariate with HCV reinfection (i.e., unadjusted model) and then to evaluate in adjusted models for covariates that were significant in the bivariate analysis and/or pre-specified in our hypotheses (i.e., multivariate adjusted models).

## Results

### Main characteristics of the study participants

Table 1 shows the sample characteristics. The sample was predominantly male (98.9%) and aged 41 and older (81.4%). Over half of the participants reported being unemployed at the time of the survey (65.2%) and had been incarcerated at least once (61.0%) in their lifetime. Among the non-injection behavioral variables, alcohol use in the past month was uncommon, with over one-third reporting never using alcohol (37.2%). Conversely, about two-thirds of respondents reported using non-injection drugs in the previous month (64%). Most participants reported no needle sharing or use of pre-used syringes in the past month (82.2% and 78.2%, respectively). About 20% of individuals reported injecting with different PWID regularly as opposed to using alone or with the same PWID. One-third of participants reported injecting several times per month in the past month, and about 21.2% reported that they regularly injected drugs every day for at least one month in the past year. Also, 33.7% of participants reported receiving drug treatment or assistance in the past year.

**Table 1:** Characteristics of the subset of participants in the IBSS study of PWID in the country of Georgia who were previously infected with HCV (N = 462) and achieved SVR after HCV treatment.

Variable	N (%)	HCV not reinfected (n = 402)	HCV Re-infected (n = 60)	P-value
**Sociodemographic**				
**City**				
Tbilisi	123(26.6)	108 (26.9)	15 (25.0)	**0.09**
Gori	57 (12.3)	54 (13.4)	3 (5.0)	
Telavi	74 (16.0)	67 (16.7)	7 (11.7)	
Zugdidi	73 (15.8)	58 (14.4)	15 (25.0)	
Batumi	35 (7.6)	27 (6.7)	8 (13.3)	
Kutaisi	36 (7.8)	31 (7.7)	5 (8.3)	
Rustavi	64 (13.9)	57 (14.2)	7 (11.7)	
**Age**				
18-24	3 (0.6)	2 (0.5)	1 (1.7)	**0.009**
25-30	12 (2.6)	9 (2.2)	3 (5.0)	
31-40	71 (15.4)	54 (13.4)	17 (28.3)	
>41	376 (81.4)	337 (83.8)	39 (65.0)	
**Age**				
Mean (sd)		49.1 (8.1)	45.7(10.1)	0.01
**Gender**				
Male	457 (98.9)	399 (99.3)	58 (96.7)	0.07
Female	5 (1.1)	3 (0.7)	2 (3.3)	
**Nationality**				
Georgian	430 (96.0)	374 (96.4)	56 (93.3)	0.26
Other	18 (4.0)	14 (3.6)	4 (6.7)	
**Education Level**				
None, primary, or secondary	275 (59.5)	236 (58.7)	39 (65.0)	0.44
Some college	31 (6.7)	29 (7.2)	2 (3.3)	
College degree	156 (33.8)	137 (34.1)	19 (31.7)	
**Employment**				
Having a permanent job	46 (10.2)	41 (10.2)	5 (8.3)	
Student/retired or disabled	20 (4.3)	18 (4.5)	2 (3.3)	
Unemployed	301 (65.2)	259 (86.0)	42 (70.0)	
Temporary Job	90 (19.5)	79 (19.7)	11 (18.3)	
**Marital status**				
Married	226 (48.9)	198 (49.3)	28 (46.7)	0.69
Divorced/Living separated from spouse	117 (25.3)	104 (25.9)	13 (21.7)	
Widow/widower	15 (3.2)	13 (3.2)	2 (3.3)	
Has never been married	104 (22.5)	87 (21.6)	17 (28.3)	
**Cohabitation**				
With spouse or partner	202 (43.7)	182 (45.3)	20 (33.3)	0.08
Alone	96 (20.8)	88 (21.9)	8 (13.3)	
With parents or relatives	133 (28.8)	109 (27.1)	24 (40.0)	
Other	25 (5.4)	21 (5.2)	4 (6.7)	
**Ever been incarcerated**				
No	177 (39.0)	155 (39.2)	22 (37.3)	0.77
Yes	277 (61.0)	240 (60.8)	37 (62.7)	
**Risky Behaviors – Alcohol Use**				
**Frequency of consumption of alcoholic beverages, such as beer, wine, vodka, and other last month**				
Never	172 (37.2)	150 (37.3)	22 (36.7)	0.42
Rarely	156 (33.8)	131 (32.6)	25 (41.7)	
Once a week	53 (11.5)	45 (11.2)	8 (13.3)	
More than once a week	58 (12.6)	54 (13.4)	4 (6.7)	
Every day	20 (4.3)	19 (4.7)	1 (1.7)	
**Risky Behaviors – Drug Use**				
**Injected drugs with the same PWIDs within the last 6 months.**				
No, alone	133 (29.3)	112 (27.9)	21 (35.0)	0.2
No, with other PWIDs	99 (21.8)	91 (22.6)	8 (13.3)	
Yes	222 (48.9)	191 (47.5)	31 (51.7)	
**Non-injection drug use in previous month**				
No	165 (35.7)	144 (35.8)	21 (35.0)	0.9
Yes	297 (64.3)	258 (64.2)	39 (65.0)	
**Injected CNS depressants within the last month**				
No	453 (98.1)	393 (97.8)	60 (100.0)	0.6
Yes	9(1.9)	9 (2.2)	0 (0.0)	
**Injected narcotic analgesics within the last month**				
No	82 (17.7)	68 (16.9)	14 (23.3)	0.2
Yes	380 (82.3)	334 (83.1)	46 (76.7)	
**Injected CNS stimulants within the last month**				
No	359 (77.7)	315 (78.4)	44 (73.3)	0.4
Yes	103 (22.3)	87 (21.6)	16 (26.7)	
**Injected opioids with continuous manner, every day during the last 12 months**				
Yes, for one month or more	176 (38.1)	146 (36.3)	30 (50.0)	**0.07**
Yes, for less than one month but more than one week	54 (11.7)	49 (12.2)	5 (8.3)	
No	216 (46.8)	195 (48.5)	21 (35.0)	
**Frequency of drugs injected within the last month**				
Once a month	30 (6.5)	27 (6.8)	3 (5.0)	0.2
Several times a month	153 (33.1)	126 (31.7)	27 (45.0)	
Once a week	40 (8.7)	37 (9.3)	3 (5.0)	
2-3 times a week	101 (21.9)	93 (23.4)	8 (13.3)	
Once a day	98 (21.2)	85 (21.4)	13 (21.7)	
Several times a day	36 (7.8)	30 (7.5)	6 (10.0)	
**The last time injected drugs, reused needle/syringe/butterfly needle**				
No	174 (40.3)	157 (41.6)	17 (30.9)	0.13
Yes,	258 (59.7)	220 (58.4)	38 (69.1)	
**In the past month reused needle/syringe/butterfly needle**				
No	351 (82.2)	309 (82.6)	42 (79.2)	0.5
Yes	76 (17.8)	65 (17.4)	11 (20.8)	
**During the last 12 months received any treatment or specific assistance for being a drug user**				
No	276 (66.3)	243 (67.5)	23 (41.1)	0.21
Yes	140 (33.7)	117 (32.5)	35 (58.9)	
**Frequency of syringe use filled in beforehand** d**uring the last month**				
Yes	98 (21.8)	84 (21.5)	14 (24.1)	0.6
No	351 (78.2)	307 (78.5)	44 (75.9)	
**Received any of the below listed products and/or information for free in Georgia during the last year - Syringe/needle/butterfly needle**				
No	167 (37.2)	146 (37.3)	21 (36.2)	0.86
Yes	282 (62.8)	245 (62.7)	37 (63.8)	
**Normally inject drugs – Street**				
No	444 (96.1)	390 (97.1)	54 (90.0)	**0.02**
Yes	18 (3.9)	12 (3.0)	6 (10.0)	
**Normally inject drugs – Apartment**				
No	70 (15.2)	60 (14.9)	10 (16.7)	0.7
Yes	392 (84.8)	342 (85.1)	50 (83.3)	
**Normally inject drugs -Automobile**				
No	377 (81.6)	332 (82.6)	45 (75.0)	0.2
Yes	85 (18.4)	70 (17.4)	15 (25.0)	
**Normally inject drugs-Entrance Hall**				
No	452 (97.8)	395(98.3)	57 (95.0)	0.13
Yes	10 (2.2)	7 (1.74)	3 (5.0)	
**Normally inject drugs -Non-residential space (garage, basement, attic, elevator, construction, abandoned house, ruins)**				
No	418 (90.5)	365 (90.1)	53 (88.3)	0.5
Yes	44 (9.5)	37 (9.2)	7 (11.7)	
**Normally inject drugs – Open space (forest, riverbank, seashore)**				
No	430 (93.1)	377 (93.8)	53 (88.3)	0.2
Yes	32 (6.9)	25 (6.2)	7 (11.7)	

### Reinfection rates

The reinfection rate was 13% (n = 60) among PWID in the 2022 IBBS study. The reinfection rate varied across cities, ranging from 25% in Tbilisi and Zugdidi to 5% in Gori.

### Risk for reinfection by covariates

Table 2 shows factors associated with HCV re-infection. In the unadjusted models, increasing age resulted in a decrease in the odds of HCV reinfection OR: 0.96 [95% CI: 0.93, 0.99].

**Table 2:** Risk factors for HCV reinfection among previously HCV-infected PWID in the 2022 IBSS study among PWID in the country of Georgia.

Variable	Unadjusted OR (95% CI)	Adjusted Odds Ratio (95% CI)
**City**		
Tbilisi	–	
Gori	0.40 [0.09, 1.28]	
Telavi	0.75 [0.28, 1.88]	
Zugdidi	1.86 [0.85, 4.10]	
Batumi	2.13 [0.79, 5.46]	
Kutaisi	1.16 [0.36, 3.27]	
Rustavi	0.88 [0.32, 2.23]	
**Age**		
18-24	4.32 [0.38, 48.74]	
25-30	2.88 [0.74, 11.08]	
31-40	2.72 [1.43, 5.14]	
41+	**–**	
**Age**	**0.96 [0.93, 0.99]**	
**Gender**		
Male	0.21 [0.03, 1.33]	
Female	–	
**Nationality**		
Georgian	0.52 [0.16, 1.64]	
Other	–	
**Education Level**		
None, primary, or secondary	–	
Some college	0.42 [0.07, 1.46]	
College degree	0.84 [0.46, 1.49]	
**Employment**		
Have a permanent job	–	
Student/retired or disabled	0.91 [0.12, 4.68]	
Unemployed	1.33 [0.54, 4.02]	
Temporary Job	1.14 [0.39, 3.83]	
**Marital status**		
Married	–	
Divorced/Living separated from the spouse	0.88 [0.43, 1.75]	
Widow	1.09 [0.16, 4.22]	
Never been married	1.38 [0.71, 2.63]	
**Cohabitation**		
With a spouse or partner	1.21 [0.53, 3.02]	0.92 [0.39, 0.92]
Other	2.10 [0.52, 7.34]	2.06 [0.49, 7.58]
With parents or relatives	**2.42 [1.08, 6.00]**	1.58 [0.66, 4.11]
Alone	–	
**Ever been incarcerated**		
No	–	
Yes	1.09 [0.62, 1.94]	
**Frequency of consumption of alcoholic beverages, such as beer, wine, vodka,and other last month**		
Never	–	
Rarely	1.30 [0.70, 2.43]	
Once a week	1.21 [0.48, 2.81]	
More than once a week	0.51 [0.14, 1.39]	
Everyday	0.36 [0.02, 1.87]	
**Non-injection drug use in previous month**		
No	–	
Yes	0.92 [0.43, 2.20]	
**Injected drugs with the same PWIDs within the last 6 months.**		
No, alone	–	
No, with other PWIDs	0.47 [0.19, 1.07]	
Yes	0.87 [0.48, 1.60]	
**Non-injection drug use in previous month**		
No	–	
Yes	0.92 [0.43, 2.20]	
**Injected CNS depressants within the last month**		
No	–	
Yes	–	
**Injected narcotic analgesics within the last month**		
No	–	
Yes	0.67 [0.36, 1.32]	
**Injected CNS stimulants within the last month**		
No	–	
Yes	1.32 [0.69, 2.40]	
**Injected opioids with continuous manner, every day during the last 12 months**		
No	–	
Yes, for one month or more	1.91 [1.06, 3.51]	**2.26 [1.06, 5.01]**
Yes, for less than one month but more than one week	0.95 [0.30, 2.46]	1.10 [0.29, 3.46]
**Frequency of drugs injected within the last month**		
Once a month	–	
Several times a month	1.93 [0.62, 8.48]	
Once a week	0.73 [0.13, 4.21]	
2-3 times a week	0.77 [0.21, 3.72]	
Once a day	1.38 [0.41, 6.32]	
Several times a day	1.80 [0.43, 9.19]	
**The last time injected drugs, reused needle/syringe/butterfly needle**		
No	–	
Yes	1.6 [0.88, 2.99]	
**In the past month reused needle/syringe/butterfly needle**		
No	–	
Yes	1.25 [0.58, 2.47]	
**During the last 12 months received any treatment or specific assistance for being a drug user**		
No	–	
Yes	1.45 [0.81, 2.56]	
**Frequency of syringe use filled beforehand** d**uring the last month**		
No	**–**	
Yes	1.16 [0.59, 2.17]	
**Received syringe/needle/butterfly needle**		
No	–	
Yes	1.05 [0.59, 1.86]	
**Normally inject drugs – Street**		
No	–	
Yes	3.61 [1.22, 9.71]	**8.08 [2.13, 30.98]**
**Normally inject drugs – Apartment**		
No	–	
Yes	0.88 [0.44, 1.92]	
**Normally inject drugs – Automobile**		
No	–	
Yes	1.58 [0.81, 2.94]	
**Normally inject drugs – Entrance hall**		
No	–	
Yes	2.97 [0.63, 11.0]	
**Normally inject drugs -Non-residential space (garage, basement, attic, elevator, construction, abandoned house, ruins)**		
No	–	
Yes	1.30 [0.51, 2.91]	
**Normally inject drugs -Open space (forest, riverbank, seashore)**		
No	–	
Yes	1.99 [0.76, 4.61]	
**Normally inject drugs – Wherever it is possible**		
No	–	
Yes	0.57 [0.13, 1.65]	

Only two variables were statistically significant in the adjusted models: injecting drugs every day in the past year and injecting on the street (i.e., public injecting). For the former, the odds of reinfection among those reporting injections every day for at least a month in the past year were 2.26 times the odds of reinfection among those who did not inject every day for any period [95% CI: 1.06, 5.01].Those reporting public injecting, had an odds of reinfection that was over 8 times that of those reporting not injecting regularly in the street [95% CI: 2.13, 30.98]. Cohabitation was significant in the bivariate analysis but not in the adjusted model; those living with parents had almost twice the odds of reinfection than those living alone in the unadjusted model and 1.58 times the odds in the adjusted model.

## Discussion

In this study, we estimated reinfection rates and examined factors associated with HCV reinfection among PWID in Georgia who had previously undergone treatment through the country’s HCV elimination program. We estimated a high HCV reinfection rate of 13%, varying by city from 5% in Gori to 25% in Kutaisi and Tbilisi. These rates are on the high end of reinfection rates among PWID estimated in other contexts, suggesting a need for targeted interventions to prevent reinfections [10–14]. Furthermore, we identified two variables that were strongly associated with HCV reinfection in our population: frequency of injections and street-based injecting (i.e., public injecting). Previous research found public injecting to be associated with infection, but no studies addressed the role of public injecting on reinfection that we are aware of [21].

In the primary analysis, we found that injecting every day for at least one month in the past year resulted in more than twice the odds of reinfection compared with not injecting every day for any period. This result underscores the importance of drug treatment programs in preventing ongoing HCV transmission in the community, especially given that almost half of the sample received no drug treatment or assistance of any kind in the past year. Previous work found that those who did not resume drug injection, at least initially, post-treatment, have a lower risk of reinfection [18]. Furthermore, the period immediately post-treatment may be an especially risky period [19]. Although everyone in our study was a current PWID, our results suggest that decreasing the frequency of drug use after treatment may be able reduce ongoing risk for reinfection. Further work should evaluate the temporal impact of injection frequency post-treatment on reinfection among PWID in the EECA region.

Street injection (i.e., public injecting) was also associated with a higher odd of HCV reinfection. Specifically, individuals who reported street-based injections were 8 times more likely to be reinfected with HCV than those who did not report street-based injections. This suggests that unsafe injection environments, peer influence, or insufficient support programs may increase reinfection risk. For example, unemployment may be associated with street-based injections. We found that those who were unemployed at the time of the survey had 33% higher odds of reinfection compared with those who were employed. However, the association was not significant in this study, but other studies have shown a significant association between unemployment and HCV infection [22]. Furthermore, unemployment has been shown to be associated with HCV prevalence in the general population of Georgia [23]. Programs that address access to safe spaces for injection, unemployment, health education, and peer groups can aid in safer injection practices, including location of injection [24].

We also identified age as a significant predictor of HCV reinfection. Younger age was associated with reinfection, as observed in other studies linking youth with higher-risk injecting behaviors [25]. Harm reduction services for young PWID face significant limitations due to their design, which predominantly targets older populations, resulting in reduced accessibility and relevance for youth. Additionally, structural barriers—including age restrictions, legal constraints, and stigma—further impede young PWID from engaging with essential harm reduction interventions, highlighting the need for tailored, youth-centered approaches to effectively reduce risks in this vulnerable group [26].

This study has several limitations. Firstly, our data was cross-sectional, so we could not investigate the temporal relationship between the reported behavioral factors and HCV reinfection. For example, those reporting street injections may have only started doing so after they were already reinfected with HCV. In addition, only 18 participants reported injections on the street. Future work should aim to identify how the location that one injects might affect reinfection.

Additionally, many behavioral variables were only asked about in the past month. Those who were reinfected earlier may have reduced their alcohol or non-injection drug use after reacquiring HCV if they felt sick, for example. Secondly, because data were self-reported, there is a risk for social desirability and recall bias. Thirdly, in some of the subgroup analyses our sample size was small, which may have limited our ability to detect significant associations and resulted in larger confidence interval estimates. Lastly, the multiple variables tested in our analysis (including various injection behaviors, drug types, and frequency patterns) increase the possibility of Type I error. However, while we examined several relevant factors such as injection of CNS depressants, narcotic analgesics, and stimulants within the last month, as well as needle reuse behaviors, none emerged as significant.

## Conclusion

While the country’s Hepatitis C elimination program has shown success in treating HCV, preventing reinfection remains a significant challenge. We estimated a high reinfection rate of 13% among PWID in Georgia, suggesting a need for interventions and programs targeting reinfection. We also identified several potential predictors of reinfection, including a high injection frequency, public injecting, and young age. Based on these results, some aspects that might be considered in designing programs include the environment in which PWID injected regularly, social programs to reduce unemployment, access to OST, and the implementation of drug consumption rooms. Future work should evaluate risk factors longitudinally so that temporality between risk factors and reinfection can be established.
